# Understanding a Potential Role for the NLRP3 Inflammasome in Placenta‐Mediated Pregnancy Complications

**DOI:** 10.1111/aji.70077

**Published:** 2025-04-22

**Authors:** Chloe G. Moss, Mark R. Dilworth, Lynda K. Harris, Sally Freeman, Alexander E. P. Heazell

**Affiliations:** ^1^ Maternal and Fetal Health Research Centre Division of Developmental Biology and Medicine University of Manchester Manchester UK; ^2^ Manchester Academic Health Science Centre Manchester University NHS Foundation Trust Manchester UK; ^3^ Department of Obstetrics and Gynaecology Olson Center for Women's Health University of Nebraska Medical Centre Omaha USA; ^4^ Division of Pharmacy and Optometry University of Manchester Manchester UK

**Keywords:** chronic histiocytic intervillositis, DAMPs, foetal growth restriction, NLRP3 inflammasome, sterile inflammation, villitis of unknown aetiology

## Abstract

Stillbirth affects approximately 2 million pregnancies annually and is closely linked to placental dysfunction, which may also present clinically as foetal growth restriction (FGR) or pre‐eclampsia (PE). Placental dysfunction can arise from a range of insults, including the inflammatory conditions villitis of unknown aetiology (VUE) and chronic histiocytic intervillositis (CHI). Despite ample research regarding the pathophysiology of placental dysfunction, the literature surrounding placental inflammation is more limited, with no currently established treatments. In the absence of infection, placental inflammation is hypothesised to be stimulated by damage‐associated molecular patterns (DAMPs), known as sterile inflammation. The NLRP3 inflammasome, a protein scaffold that unites within the cytosol of cells, is a proposed contributor. The NLRP3 inflammasome is dysregulated in numerous diseases and has shown evidence of activation through the sterile inflammatory pathway via DAMPs. Studies have demonstrated the upregulation of the NLRP3 inflammasome and its components in placentally‐mediated pregnancy pathologies. However, the link between placental dysfunction seen in these disorders and the NLRP3 inflammasome is not yet firmly established. This manuscript aims to review the evidence regarding placental inflammation seen with placental dysfunction, discuss its association with the NLRP3 inflammasome, and identify potential therapeutic interventions for this pathological inflammatory response.

## 1 Introduction

Stillbirth is defined by the World Health Organization as the death of a foetus in utero at or after 28 weeks gestation [1], with a worldwide incidence rate estimated at 1.9 million in 2021 [2]. Placental dysfunction is a major cause of stillbirth, and is often associated with foetal growth restriction (FGR) and pre‐eclampsia (PE). FGR occurs in 5%–10% of pregnancies [3], equating to around 30 million affected foetuses each year [4]. FGR is estimated to be responsible for 30%–43% of stillbirth cases in high‐resource settings [5], making it the second leading cause of perinatal mortality [3], and increasing the risk of perinatal morbidity 5–30 times [4, 6]. The placental pathologies underlying stillbirth and FGR include vascular, genetic, and inflammatory causes [3]. Inflammatory placental disorders include, villitis of unknown aetiology (VUE) [7] and chronic histiocytic intervillositis (CHI) [8]. However, even when these conditions have not been diagnosed, excess placental immune cells are seen in cases of FGR and stillbirth [7, 9]. Due to the absence of infection in these cases [7, 8], the inflammation seen is theorised to be induced through the sterile inflammatory pathway. The NLRP3 inflammasome, an intracellular protein complex, is activated via damage‐associated molecular pattern (DAMP)‐mediated stimulation [10, 11]. As a growing body of literature implicates its involvement in inflammatory placental pathologies [12, 13, 14, 15, 16], it is of interest to elucidate its role within these conditions. This literature review aims to evaluate the evidence regarding the causes of inflammation in placental pathologies, with a focus on FGR, discuss their association with the NLRP3 inflammasome and identify potential therapeutic entities.

## 2 The Placenta

The placenta is an extraembryonic organ that is crucial for successful foetal growth and development as it performs vital functions to maintain a healthy pregnancy. The placenta manages hormonal regulation, immunological acceptance, the delivery of oxygen and nutrients to the foetus, and the removal of waste products; the latter two activities are accomplished via the placental villi [17]. Placental development is tightly regulated to perform these functions efficiently. Generation of the trophectoderm (TE) layer initiates placental formation, developing from the blastocyst 4–5 days post‐conception, then interacting with the uterine luminal epithelium 2–3 days later to instigate implantation [17]. Post‐implantation, trophoblast lineages form from the TE, commencing with cytotrophoblasts 8 days post‐fertilisation [17]. Around 2 days later, the generation of the primary placental villi ensues, which later mature into secondary and subsequently tertiary villi to allow for maternal–foetal communication, together with the formation of the outer syncytiotrophoblast layer [17]. Appreciation of placental structure and cellular composition allows a deeper understanding of the dysfunction that occurs in pregnancy complications.

## 3 Foetal Growth Restriction

FGR is a pregnancy complication defined by a foetus’ failure to achieve its growth and developmental potential in utero, usually due to impaired placental function [3, 4, 18]. Historically, FGR has had various definitions, which have, to some extent, been influenced by tools to monitor foetal growth in utero. Previously, small for gestational age (SGA) was used interchangeably with FGR, thus, infants whose birthweights/measurements were <10th centile were classified as FGR/SGA [19], until it was suggested that SGA infants were otherwise healthy aside from low birthweight [20], and therefore should not be categorised as FGR. In 2016, a Delphi consensus established agreed criteria for FGR: the estimated foetal weight (EFW) and foetal abdominal circumference should be <3rd centile, congenital/chromosomal abnormalities should be absent, with the gestational threshold differentiating early and late FGR being 32 weeks [18]. The increased specificity of these criteria better identifies adverse neonatal outcomes, which are themselves more strongly related to placental dysfunction.

FGR can cause both short and long‐term difficulties, with foetal death in utero being the most significant short‐term complication [3, 5]. Long‐term health consequences can occur due to low birthweight as a result of developing in an adverse pregnancy environment [21]. This is reflected in FGR‐affected infants, who show evidence of compromised cognitive development postnatally, including emotional, behavioural and social difficulties [4], alongside an increased risk of neurological disorders, such as cerebral palsy [3, 6, 18, 19]. Furthermore, individuals classed as FGR suffer from an increased risk of cardiovascular diseases and endocrine complications later in life [3, 4, 18, 19]. To date, treatments for FGR have been limited and unsuccessful, with delivery of the foetus being the only current option, subject to its gestational age and health [6]. Ineffective treatments include bed rest, nutritional supplementation, maternal oxygen therapy, plasma volume expansion [3, 6] and administration of sildenafil, all of which have been trialled with little success [3, 6, 22]. The studies suggest that increasing nutrient or oxygen concentration in maternal blood or altering perfusion of the intervillous space does not improve outcomes. Treatment with antenatal corticosteroids has demonstrated an improvement in foetal pulmonary conditions associated with FGR [6], but outcomes are inconsistent, and in one study, contrarily caused decreased foetal growth [23], implying the necessity for further research before use, as verified more recently [24]. An improved understanding of the underlying pathophysiological processes in FGR is required to develop effective therapeutic or preventative strategies.

Various placental abnormalities are associated with FGR, including a reduction in size [6] and abnormal development of the terminal villi [20], and consequently, a reduction in nutrient and oxygen delivery which has numerous implications for both mother and infant. This placental dysfunction can be attributed to foetal, maternal, and uteroplacental origins [3, 6]. Foetal factors include, but are not limited to, chromosomal abnormalities, genetic syndromes and intrauterine infections [3]. Maternal factors include clinical diseases (such as hypertensive pregnancy diseases, autoimmune diseases, thrombophilia, severe anaemia and renal diseases, hyperhomocysteinemia, restrictive pneumopathies, insulin‐dependent diabetes mellitus with vasculopathy and congenital cyanotic heart diseases) nutritional disorders and drug use [3]. Uteroplacental factors are the greatest contributors to FGR [20], encompassing inadequate placentation, described as a reduction of trophoblast invasion and remodelling of the uterine spiral arteries [3]. Likewise, maternal vascular disease, associated with diminished uteroplacental perfusion, falls within this category, as the prevailing cause of FGR, instigating 25%–30% of cases [3]. Maternal vascular disease encompasses both pre‐pregnancy maternal systemic vascular diseases, such as chronic hypertension [25, 26], as well as impaired uterine vascular function during pregnancy (increased vasoconstriction and reduced relaxation of myometrial small arteries) [27, 28], both leading to reduced uteroplacental perfusion and adverse pregnancy outcomes.

PE is another known cause of perinatal morbidity and mortality and is described as a de novo hypertensive pregnancy disorder regularly accompanied by proteinuria [29]. It is characterised by both inadequate trophoblast invasion and incomplete uterine spiral artery remodelling during placentation [29], similarly to cases of FGR [30], alongside an imbalance in circulating angiogenic biomarkers resulting in maternal endothelial dysfunction [29]. It is associated with multisystemic damage, including pulmonary oedema, neurological complications, renal, cardiac, haematologic and hepatic dysfunction, and occasionally results in eclampsia and death [29]. Contributors of placental dysfunction in PE can promote FGR development, frequently seen in PE patients [29]. Similarly to FGR, the leading treatment is delivery of the foetus and placenta; however, aspirin has been proposed to reduce the risk of PE in some women [29].

The structural irregularities intrinsic to FGR and PE lead to placental dysfunction and subsequent pathophysiological changes. For example, impaired spiral artery remodelling may alter placental size and or shape, and perfusion of the intervillous space, thereby reducing the number of villi and their vascularity, leading to reduced foetal blood flow and ultimately impairing diffusion [31, 32]. This can consequently diminish nutrient transfer [30] and reduce oxygen diffusion across the placenta, leading to foetal hypoxia [33]. Furthermore, the resulting compensatory responses can lead to preferential delivery of blood to the foetal brain, depleting both glycogen and adipose tissue stores and resulting in maintenance of normal brain growth, at the expense of abdominal size [20]. This so‐called brain‐sparing effect is a phenotype seen in many FGR affected infants.

Systemic and placentally localised inflammation is a notable characteristic of FGR and is signified by elevated levels of pro‐inflammatory cytokines and chemokines [34]. Additionally, studies show increased numbers of CD163^+^ macrophages in the placentas from both live and stillborn infants with FGR [7], as well as those experiencing decreased foetal growth [9]. Placental pathophysiological anomalies associated with FGR are likely responsible for the exacerbated inflammatory response seen in this condition [34]; however, this may also occur through the development of de novo inflammatory disorders of pregnancy.

## 4 Inflammatory Disorders of the Placenta

Inflammation of the placenta may occur due to infections such as cytomegalovirus, zika virus, and SARS‐CoV‐2 which cause villitis or intervillositis in some cases [35, 36, 37]. Alternatively, inflammatory disorders including CHI and VUE can occur in the absence of infection, so‐called sterile inflammation [7, 8, 38].

CHI, sometimes referred to as massive chronic intervillositis or massive perivillous histiocytosis [39], is rare [40, 41], occurring in six out of every 10 000 pregnancies post 12 weeks of gestation [8]. However, it has a recurrence rate between 25% and 100% and can develop during any trimester [8, 42]. CHI is associated with a higher risk of pre‐term birth, miscarriage, stillbirth, PE, and FGR [8, 40]. It is characterised by maternal histiocytes, specifically CD68^+^ macrophages, invading the intervillous space, sometimes accompanied with fibrinoid deposition and trophoblast necrosis [8, 40–43]. Although the aetiology of CHI is incompletely understood, it is theorized to result from adverse complement activation, with B and Treg lymphocytes from the maternal immune system aberrantly reacting to foetal paternal antigens [8, 40], leading to placental inflammation. As this is comparable to an antibody‐mediated organ transplant rejection response, C4d staining was utilised to affirm this. Healthy placentas lacked evidence of C4d immunostaining, whilst those with CHI demonstrated immunoreactivity within syncytiotrophoblast [43]. However, this hypothesis was questioned more recently [44], further highlighting the ambiguity of its aetiology.

VUE is one of the most frequently seen inflammatory placental diseases [45], with a prevalence of 3.7%–18.6% [45], and a high risk of recurrence, ranging from 25% to 50% [45]. VUE is identified by the infiltration of lympho‐histiocytes, more specifically CD68^+^ foetal macrophages (Hofbauer cells), CD8^+^ cytotoxic and CD4^+^ T lymphocytes into the placental villous stroma [7, 45]. An increased expression of the pro‐inflammatory cytokine IL‐2 has also been noted in VUE lesions [7]. Similarly to CHI, the failure of immune tolerance is the hypothesized aetiology of VUE [8]. More definitively, it has been described as an abnormal maternal T lymphocyte response against foetal paternal alloantigens expressed by Hofbauer cells in placental villi, similar to transplant allograft rejection [45].

Occasionally, VUE and CHI present concurrently, though CHI presents sooner and demonstrates a higher morbidity [8]. Both conditions also have associations with FGR, although it occurs more commonly and with a greater risk of stillbirth in CHI [8]. FGR is the leading adverse consequence associated with VUE, presenting in 19.2%–34.4% of cases, classifying them as high grade VUE [45]. Inversely, the risk of developing VUE is higher in women presenting with FGR, and increases in prevalence when FGR is recurrent [45]. Although VUE and CHI are strongly associated with FGR and pregnancy loss, it should be noted that a causal relationship between these conditions and pregnancy outcome has not yet been established.

Both CHI and VUE are asymptomatic [8] and lack evidence of infection [7, 42], relying solely on investigations of postnatal placental histopathology for diagnosis [7, 8], therefore remedial options are only proposed for subsequent pregnancies. Furthermore, the recurrence rate of CHI and VUE may only be 25% in some cases [8, 45], and it is therefore unclear whether trials for treatments were effective or if there was simply an absence of these pathologies independent of any treatment. Potential treatments investigated to date include aspirin, low molecular weight heparin, steroids (such as prednisolone), IL‐1RA, colchicine, and hydroxychloroquine [8, 45, 46]; however, due to a lack of high‐quality intervention studies, none have been definitively recommended to treat either condition. The limited literature surrounding these pathologies creates challenges for their management and a better understanding of their underlying inflammatory pathophysiology could enable directed exploration of anti‐inflammatory therapeutics.

## 5 Sterile Inflammation in the Placenta

Physiological inflammation plays an important role throughout gestation, including maintenance of a healthy pregnancy and eventually parturition [47]. Inflammation first occurs during implantation and placentation, as the invasive processes simulate an “open wound”, inducing an inflammatory response to repair the uterine epithelium and remove cellular debris [48]. This continues throughout the first trimester and early second trimester, and is described as the pro‐inflammatory phase [48]. Finally, infiltration of immune cells are required for uterine contractions and delivery of both baby and placenta [48]. However, these processes are not indicative of the pathological placental inflammation seen in FGR and stillbirth.

Atypical placental inflammation has been associated with numerous pregnancy complications [49] and increases the risk of stillbirth and FGR [50]. High‐risk pregnancies associated with reduced foetal movements have demonstrated increased expression of the pro‐inflammatory cytokines IL‐1β, IL‐18, and IL‐1Ra, and a decrease in anti‐inflammatory cytokine IL‐10 in the placenta and maternal serum [51]. Additionally, foetuses exhibiting a decreased growth rate in the third trimester show an increase in the number of placental macrophages, which may elude to a pro‐inflammatory change in late onset FGR [9]. The link between placental dysfunction and inflammation has recently been attributed, in part, to the NLRP3 inflammasome [47, 52, 53].

**FIGURE 1:**
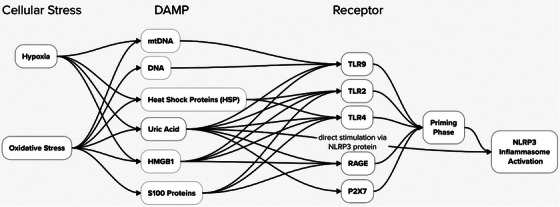
The different pathways cellular stressors act through to cause NLRP3 inflammasome priming and activation, including the specific DAMPs they stimulate and the associated receptors they bind to. This diagram summarises information from various publications [55, 56, 57, 58, 59, 60, 61, 62]. Figure created using Mural diagramming software.

Sterile inflammation describes an inflammatory response in the absence of an invading pathogen or infection [11], with DAMPs released by pathological conditions being the suggested trigger [11] (Figure 1). This inflammatory type has been previously discussed in placental conditions such as FGR, PE, CHI, and VUE, and therefore, the inflammation seen may theoretically be stimulated by this pathway. For example, immobility stress in mice resulting in FGR was associated with increased TNF‐α expression (an oxidative stress marker) in murine placentas [54], implying the contribution of the sterile inflammatory pathway in pregnancy complications.

It is hypothesised that placental damage/insult induces release of DAMPs, instigating the sterile inflammation seen in FGR and stillbirth [56]. This is supported by a study which subjected placental explants to hypoxia, oxidative, or nitrative stress. Following hypoxia or oxidative stress exposure, increased uric acid, HMBG1, and S100 concentrations were demonstrated in the culture medium, with oxidative stress also increasing cell‐free foetal DNA (cffDNA) release, and hypoxia increasing HSP70 levels [56]. These environments also increased the production and release of pro‐inflammatory cytokines including IL‐1β, from placental explants subject to oxidative stress [63]. Furthermore, cytokine levels were reduced after addition of the caspase‐1 inhibitor, FMK002 [56], a known NLRP3 inflammasome inhibitor. However, after validating these findings via immunohistochemistry (IHC), only S100A8 protein expression increased in hypoxic placental tissue, highlighting the necessity for additional investigations to understand how DAMP signalling is mediated within the placenta.

Other studies have explored DAMP levels in the context of pregnancy complications, with findings revealing an increase in placental uric acid levels in pregnancies that experienced reduced foetal movements, which were further elevated still in pregnancies that also experienced a decreased foetal growth rate, defined as a drop in ≥25 centiles between the estimated foetal weight in the third trimester, determined via ultrasound scanning, and actual birthweight [9]. In the same study, an increase in S100A8 and HSP70 protein levels were additionally discovered in the maternal serum of pregnancies that exhibited a decreased foetal growth rate [9]. More recently, elevated S100A9 protein expression was observed in endometrial tissue of women with recurrent pregnancy loss [38], which supports the hypothesis that DAMP release contributes to sterile inflammation in pregnancy disorders. To appreciate how these DAMPs mediate inflammation, their role within the NLRP3 inflammasome must be discussed.

## 6 The NLRP3 Inflammasome

The innate immune system is the body's first line of defence against harmful stimuli and has numerous components, including the inflammasome—a multimeric protein complex that unites within the cytosol [10, 11]. These intracellular scaffolds contain pattern recognition receptors (PRRs) which recognise DAMPs and pathogen‐associated molecular patterns (PAMPs), specific to the type of inflammasome, inducing a cascade of events, and ultimately an inflammatory response [10, 11, 64]. Inflammasomes are categorised into families defined by the unique PRR they are assembled with [11]; these are: nucleotide‐binding oligomerization domain (NOD) leucine‐rich repeat (LRR)‐containing proteins (NOD‐like receptor, NLRs), including NLRP1, NLRP3, and NLRC4, and absent‐in‐melanoma‐2 (AIM2)‐like receptors, known as ALRs, and pyrin [11, 64, 65].

The NLRP3 inflammasome is currently the most researched of all inflammasomes and is found in immune cells such as macrophages/monocytes, neutrophils, lymphocytes, and dendritic cells, as well as microglia, epithelial cells, osteoblasts, and neurons [10]. Its primary role is defending the body against bacterial, viral, and fungal infections [64], and like other inflammasomes, when dysregulated it is associated with autoinflammatory and autoimmune diseases [11]. The NLRP3 inflammasome recognises PAMPs, including the lipopolysaccharide (LPS) component of bacteria, and DAMPs, such as extracellular ATP (eATP) and glucose, monosodium urate (MSU) crystals, fatty acids, amyloid deposits, cholesterol [65], HMGB1, and cffDNA [50]. DAMPs are a product of endogenous cellular stress or damage [10], and their purpose is to induce a protective inflammatory reaction.

**FIGURE 2:**
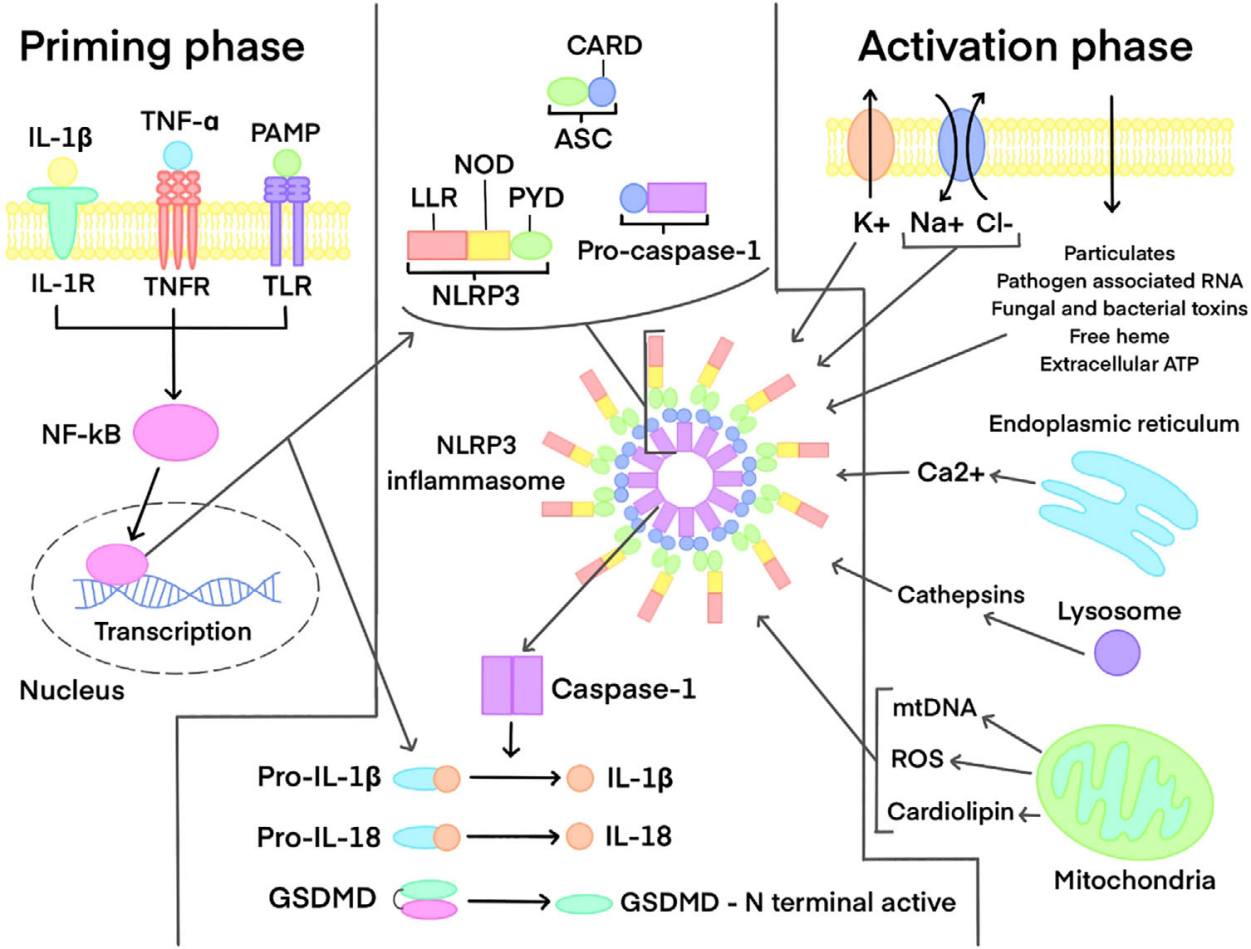
The structure of the NLRP3 trimeric protein and the components of the NLRP3 inflammasome complex. The stimuli for the priming and activation phase, as well as the subsequent downstream signalling events following NLRP3 inflammasome formation are shown. The products resulting from NLRP3 inflammasome activation are also depicted.

To understand how the NLRP3 inflammasome functions, its structure must be appreciated. The complex includes three main components (Figure 2): the NLRP3 protein, an apoptosis‐associated speck‐like adaptor protein containing a caspase‐recruitment domain (ASC), and caspase‐1 [10, 65]. Similarly to the unified complex, the NLRP3 protein consists of three segments, an N‐amino terminal pyrin domain (PYD), a central NOD, and a C‐terminal LRR domain [10, 64].

Activation of the NLRP3 inflammasome requires two steps, a priming signal and an activation signal (Figure 2) [64]. The priming phase is induced by the presence of endogenous stress markers, including cytokines (tumour necrosis factor‐α [TNF‐α] [66], IL‐1β) and microbial constituents (LPS) [64]. This stage is primarily transcriptional, although transcription‐independent pathways have recently been described [64]. Stimuli trigger the corresponding Toll‐like receptors (TLRs), IL‐1R, or TNFR which in succession activate the transcription factor nuclear factor‐κB (NF‐κB), causing NLRP3 upregulation [11] and pro‐IL‐1β expression [64, 65]. Many stimuli are thought to induce the secondary activation phase, including eATP, ionic flux (K^+^ efflux [11, 65], Ca^2+^ mobilization, Na^+^ influx, and Cl^−^ efflux), lysosomal damage releasing cathepsins into the cytosol [11, 65], free heme, particulates, pathogen‐associated RNA, and bacterial and fungal toxins [64]. Mitochondria are another suggested activator if dysregulated, via increased production of reactive oxygen species (ROS) [65] and the release of mitochondrial DNA or cardiolipin [11, 64]. Despite their varied nature, these stimuli are suggested to promote similar cellular events to trigger activation; however, their mechanisms of action are still speculative [11, 64, 65] and all pathways for NLRP3 inflammasome regulation are yet to be fully elucidated.

For inflammation to occur, DAMPs/PAMPs activate the NLR through the previously described two‐step event, causing its oligomerization and transforming into a pro‐caspase‐1 activating frame [11, 64]. Sequentially, the PYD component of both NLRP3 and ASC interact, which likewise occurs for the caspase recruitment domain (CARD) of both ASC and pro‐caspase‐1, to commence inflammasome assembly [10, 11, 64]. Once recruited into the inflammasome, pro‐caspase‐1 converts into its active form, caspase‐1, by proximity‐induced auto‐proteolytic cleavage [10, 11]. Caspase‐1 cleaves pro‐interleukin‐1β (pro‐IL‐1β), pro‐interleukin‐18 (pro‐IL‐18) and gasdermin D (GSDMD) into their biologically active forms, IL‐1β, IL‐18 [11], and NT‐GSDMD, respectively [64, 65]. IL‐1β upregulates expression of genes which induce signs of inflammation, whilst IL‐18 stimulates the production of interferon‐γ and communicates with the adaptive immune system [10, 11, 64, 65]. The N‐terminal domain of GSDMD causes pores in the plasma membrane, initiating pyroptosis, a pro‐inflammatory form of cell death, subsequently releasing DAMPs and triggering further inflammation [10, 11, 64, 65]. This mechanism of activation is accepted as the canonical pathway, and can be observed in Figure 2; non‐canonical and alternative pathways of NLRP3 activation have also been described [10, 11, 64].

NLRP3 inflammasome‐induced inflammation can transmit between cells, due to an accumulation of ASC specks within the extracellular space, which become phagocytosed by macrophages, leading to further IL‐1β production and inflammation [67]; a mechanism suggested to be important in a number of inflammatory diseases [11]. Typically, the body regulates activation of the NLRP3 inflammasome to prevent the initiation and progression of inflammatory disorders or damage to host tissue, and achieves this through several methods. Autophagy inhibits activation of the NLRP3 inflammasome by depleting endogenous stimuli; likewise, lowered mitochondrial activity reduces ROS levels, yielding similar results [65]. Similarly, type 1 interferons and CD4^+^ T cells act via caspase‐1 to reduce NLRP3 activity; however, their mechanisms are poorly understood [65]. Other known negative regulators are TRIM20, microRNAs (such as miR‐30a [68]), pyrin‐only, and CARD‐only proteins, and although they act via different mechanisms [65], they all limit inflammation.

## 7 Evidence for the NLRP3 Inflammasome in the Placenta and in Pregnancy Complications

Inflammation is necessary throughout pregnancy [47], with evidence suggesting contributions from the NLRP3 inflammasome, signified by high levels of its stimuli, TNF‐α, during implantation [48]. It has also been identified in the placenta and chorioamniotic membranes, due to the expression of its associated biomarkers, caspase‐1 and IL‐1β [47], as well as in trophoblast during the first trimester and at term [47, 69]. In addition, human first trimester trophoblast cultures reportedly secrete IL‐1β through the NLRP3 inflammasome via TLR4 signalling [70]. Both TLR2 and TLR4 are expressed by trophoblast subtypes throughout early gestation, with TLR4 continuing to be expressed in the second and third trimester by syncytiotrophoblast, Hofbauer cells, and endothelial cells [71]. An investigation of isolated Hofbauer cells from human term placentas further verified the presence of the NLRP3 inflammasome after LPS and ATP stimulation, by observing increased IL‐1β, GSDMD and caspase‐1 expression [72]. This is thought to function in the pro‐inflammatory environment during parturition [47]. Maternally, the NLRP3 inflammasome has been discovered in peripheral leukocytes, the myometrium at term [47], oocytes and the uterine endometrium preconception [73]. It has also been reported to contribute to ovarian ageing and if inhibited, can extend fertility in female mice [73].

Despite its role in healthy gestation, the NLRP3 inflammasome is proposed to contribute to several pregnancy complications [52, 74]. Products of the NLRP3 inflammasome pathway have been identified in pregnancy complications. Evidence of GSDMD has been identified in the amniotic fluid and chorioamniotic membranes from individuals who underwent spontaneous preterm birth [75], and women who experienced recurrent miscarriages exhibit upregulation of IL‐1β and IL‐18, in placental tissue and decidua, respectively [76, 77]. Therefore, if generated through the NLRP3 inflammasome pathway, these products likely contribute to pregnancy complications further by promoting pyroptosis and stimulating additional inflammation [10, 11, 64, 65], exacerbating existing complications.

In general, the published literature describes increased expression of NLRP3 inflammasome constituents in disorders of pregnancy, summarised in Table 1. These nine studies provide evidence of NLRP3 upregulation in maternal serum, decidual tissue and the trophoblast of placental tissue. Experiments utilising NLRP3 inflammasome inhibitors further confirmed these results, with similar conclusions drawn from murine models. Furthermore, an NLRP7 inflammasome study discovered a significant increase in PYCARD (ASC‐speck) protein expression in FGR‐affected placentas [78]. Although differing from the NLRP3 inflammasome, both require the ASC‐speck for assembly [11], reflecting results seen in NLRP3 studies. Further IHC analysis located PYCARD within trophoblast, demonstrating its presence at low levels within normal placental tissue [78], and affirming inflammasome expression during healthy pregnancies. Despite this, the role of NLRP3 inflammasome upregulation in FGR is uncertain, with inconsistent results between studies (Table 1).

**TABLE 1:** A summary of the literature available on the expression of the NLRP3 inflammasome in inflammatory disorders of pregnancy.

Author/Date	Tissue/Cell type	Number of samples	Experimental methods	Findings
**FGR/PE studies**				
Stødle et al., 2018 [69]	Human placental tissue	Healthy pregnant controls *n* = 13, PE with FGR *n* = 12, PE without FGR *n* = 11.	IHC	No difference in syncytiotrophoblast NLRP3 expression between women with PE alone, or combined with FGR, compared to healthy controls. No difference in expression between PE patients with or without FGR.
Silva et al., 2020 [12]	Decidua basalis tissue; paraffin embedded	Healthy control pregnancies *n* = 41, PE with FGR *n* = 26, PE without FGR *n* = 18.	IHC was conducted on decidual samples which then underwent automated quantification for protein expression analysis.	NLRP3 was expressed in extravillous trophoblasts, maternal leukocytes, decidual stromal cells, and endothelial cells. NLRP3 and IL‐1β expression intensity and density were increased in PE without FGR, compared to normal pregnancies and those with PE and FGR, specifically in areas containing trophoblasts and maternal leukocytes.
Xie et al., 2009 [13]	Human neutrophils	*n* = 25 for all groups: EOPE, LOPE, normotensive FGR (NFGR), healthy control pregnancies matched to each group of pathologies, non‐pregnant women.	Maternal blood was collected, and neutrophils were isolated for RT‐qPCR analysis of mRNA expression and flow cytometric analysis of protein expression.	Those with PE demonstrated an increase in TLR2 and TLR4 mRNA and protein expression, compared with matched healthy pregnancy controls. IL‐1β mRNA expression was also increased in EOPE compared to non‐pregnant controls. Those with EOPE also demonstrated upregulated mRNA levels of TLR2, TLR4 and NF‐kB compared to NFGR women.
Alfian et al., 2022 [14]	Human placental tissue	*n* = 25 for both groups: FGR, healthy pregnant controls.	Fluidigm Biomark Array (High‐throughput real‐time PCR), validated by RT‐qPCR, WI, and IF.	FGR‐affected placentas demonstrated a significant increase in NLRP3, IL‐1β, CASP1, NFKB, and TLR2 mRNA expression, alongside NLRP3 protein levels. A 2‐fold increase in NLRP3 mRNA expression was observed in pre‐term FGR compared to pre‐term controls, and a 3.7‐fold increase for term FGR compared to term controls. NLRP3 was localised to syncytiotrophoblast, villous cytotrophoblasts, stromal cells and endothelial cells surrounding the foetal capillaries.
	Murine FGR (LPS‐induced inflammation) model; placental tissue	*n* = 4–6 for both groups: FGR placentas, healthy pregnant controls.	IHC	An increase in NLRP3 and caspase‐1 protein levels were observed.
Pan et al., 2021 [15]	Human placental tissue	Severe PE *n* = 48, healthy pregnant controls *n* = 20.	RT‐qPCR, WI, IHC, and IF.	Significant increase in mRNA and protein expression of NLRP3, caspase‐1, and GSDMD in patients with severe PE. Only IL‐1β mRNA expression was increased in severe PE. All proteins were localised to villous trophoblasts.
Lv et al., 2023 [16]	Human umbilical cord samples and cord sera	*n* = 8 for both groups: PE, healthy pregnant controls.	WI was conducted on umbilical tissue. ELISAs were conducted on the serum.	Both umbilical cord serum and tissue exhibited a significant upregulation of IL‐1β and IL‐18 in PE patients. Protein expression of NLRP3, caspase‐1, IL‐1β, and IL‐18 were upregulated in umbilical vessels.
**Studies on other pregnancy disorders**				
Lu et al., 2019 [79]	Human peripheral blood monocytes (PBMCs), decidual tissue	*n* = 30 for both groups: women who experienced RSM, healthy controls.	Methodology for PBMCs: RT‐qPCR and ELISA. The methodology for decidual tissue analysis was unspecified.	Increased NLRP3 mRNA expression in PBMCs and decidual tissue from RSM women. Increased caspase‐1 activity, and IL‐18 and IL‐1β levels were also seen in PBMCs from RSM women.
	Murine splenic tissue, murine embryos	*n* = 10 for both splenic tissue groups: pregnant RSM models, healthy control pregnancies. *n* = 10 for both treatment groups: pregnant RSM model, pregnant with inhibitor.	RT‐qPCR and ELISA were conducted on splenic tissue. Treatment with the caspase‐1 inhibitor YVAD was used in murine RSM models.	NLRP3 mRNA expression, caspase‐1 activity, and IL‐1β and IL‐18 levels were significantly upregulated in splenic tissue from RSM mice, compared to controls. RSM mice treated with YVAD demonstrated a reduced embryo absorption rate compared to the untreated RSM mice.
Mattuizzi et al., 2024 [46]	Human placental tissue	CHI *n* = 18, healthy pregnant controls *n* = 6, three pregnant patients with a history of CHI who were trialling preventative medications.	RNA seq of placental tissue. The combination of medications tested included anakinra and colchicine (inflammasome inhibitors); placentas were analysed using IHC.	An increase in mRNA expression of NLRP3, PYCARD, CASPASE, IL‐1β, and IL‐18 was exhibited in CHI affected placentas. Treatment with the combination of medications significantly downregulated placental NLRP3 and PYCARD protein levels.
Zhang et al., 2019 [80]	Murine pancreatic tissue	GDM mouse model *n* = 8 for each treatment group.	Treatment groups: Administered water (vehicle), low and high dose astragaloside IV (AS‐IV) (NLRP3 inflammasome inhibitor); pancreatic tissue analysed using WI.	Protein levels of IL‐1β, caspase‐1, p10, and NLRP3 were elevated in pancreatic tissue from the GDM mouse model. Treatment with AS‐IV reduced these levels in a dose‐dependent manner.

Abbreviations: CHI, chronic histiocytic intervillositis; ELISA, enzyme‐linked immunosorbent assay; EOPE, early onset preeclampsia; FGR, foetal growth restriction; GDM, gestational diabetes mellitus; IF, immunofluorescent staining; IHC, immunohistochemistry; LOPE, late onset preeclampsia; PE, preeclampsia; RSM, recurrent spontaneous miscarriage; RT‐qPCR, real‐time quantitative reverse transcription polymerase chain reaction; WI, western immunoblotting.

Discrepancies may be attributed to methodologies or sample size, for example, Stødle et al. and Alfian et al. (Table 1) observed opposing results, despite both investigating human placental tissue, with the former not detecting differences in NLRP3 protein expression in cases of PE, with or without FGR. However, a smaller sample size was utilised, with NLRP3 expression solely assessed through IHC. Using a larger sample size, Alfian et al. applied a variety of techniques, including PCR and protein imaging, allowing assessment of mRNA expression and NLRP3 inflammasome activation. They reported an increase in mRNA expression of NLRP3 inflammasome components in cases of FGR, highlighting the benefits of a more thorough evaluation. Comparably, Pan et al. (Table 1) reported similar results in a PE study, applying a similar range of methodologies and an even greater sample size. Differences in FGR birthweight criteria between studies (Stødle et al: <5th centile, Alfian et al: <10th centile) may also be responsible for the variations in results. These discrepancies confirm the need for further adequately controlled studies in this area.

Markers of sterile inflammation (DAMPs) have been investigated in context of the NLRP3 inflammasome and inflammatory disorders of pregnancy (Table 2). Overall, elevated levels of DAMPs were observed in those who experienced inflammatory pregnancy pathologies. Furthermore, treatment with DAMPs largely spurred upregulation of NLRP3 inflammasome components in a variety of tissue and cell types, including placental explants and human umbilical vein endothelial cells (HUVECs). Again, these results were reflected in complementary murine studies. Gomez‐Lopez and coworkers summarised their research, which showed that HMGB1 is present in amniotic fluid of women who experienced preterm labour, initiates preterm birth in mice, and activates the NLRP3 inflammasome in chorioamniotic membranes [47]. S100B was also found to activate the NLRP3 inflammasome, causing sterile intra‐amniotic inflammation and preterm birth [47], providing further evidence to support this narrative. Despite the assortment of research on multiple DAMPs, inconsistent results are found between studies, suggesting the role of other inflammatory mediators in the pathogenesis of inflammatory disorders of pregnancy. This emphasizes the importance of further research into how these mediators impact the pathogenesis of inflammatory disorders of pregnancy.

**TABLE 2:** Summary of the literature available on sterile inflammation markers (DAMPs), their interactions with the NLRP3 inflammasome, and their presence in inflammatory disorders of pregnancy.

Author/Date	Tissue/Cell type	Number of samples	Experimental methods	Findings
Silva et al., 2020 [12]	Decidua tissue explant culture	*n* = 3.	LPS‐primed explants were treated with 200 or 2000 µg/mL synthetic cholesterol crystals, cytokine release was measured via an ELISA.	Treatment with cholesterol crystals significantly increased the release of IL‐1β.
	Decidua basalis tissue snaps frozen	Healthy control pregnancies *n* = 34, PE with FGR *n* = 27, PE without FGR *n* = 15.	Automated quantification was used to identify cholesterol crystals in decidual tissue cryosections.	Cholesterol crystals were identified in decidual tissue; however, there was no difference in the amount or distribution between normal pregnancies and PE patients alone or combined with FGR.
Shirasuna et al., 2015 [81]	NLRP3^−/−^ murine model foetal weights	*n* = 42–89 pups from each group: NLRP3^−/−^ model angiotensin‐II high‐dose, NLRP3^−/‐^ model angiotensin‐II low‐dose, WT angiotensin‐II high‐dose, WT angiotensin‐II low‐dose, vehicle.	NLRP3^−/−^ and WT mice were treated with angiotensin‐II (500 or 1500 ng/kg body weight each day from GD10‐17) to mimic the hypertension seen in PE. The vehicle (control) group received a PBS infusion. Foetal weights were measured at GD17.	NLRP3^−/‐^ mice exhibited a decreased foetal weight after high‐dose angiotensin‐II stimulation compared to the WT.
Brien et al., 2017 [50]	Primary cytotrophoblast cells	*n* = 6–9 for each group: MSU crystal stimulation, MSU crystal stimulation with inhibitor, untreated controls.	Cytotrophoblasts were treated with MSU crystals (100 µg/mL) ± a caspase‐1 inhibitor (10mM). Protein analysis was then conducted.	Stimulated cytotrophoblasts demonstrated an 18.6‐fold increase in caspase‐1 mediated IL‐1β production, which was blocked by co‐treatment with the caspase‐1 inhibitor.
	Healthy term placental explant tissue	*n* = 6–9 for each group: MSU crystal stimulation, MSU crystal stimulation with inhibitor, untreated controls.	Explant tissue was treated with MSU crystals (100 µg/mL) ± a caspase‐1 inhibitor (10mM). Protein analysis and WI was then conducted.	MSU crystal‐treatment of placental explants induced IL‐1β secretion, which was blocked by co‐treatment with the caspase‐1 inhibitor. MSU treatment also increased caspase‐1 activity within the supernatant.
	Rat placental tissue, foetuses	Dams/litters *n* = 6 for each group: three different doses of MSU crystals + potassium oxonate (uricase inhibitor) in pregnant dams, PBS‐treated pregnant controls.	Pregnant rats were treated with MSU crystals (250, 500, or 100 µg/kg/12 h from G18‐G21). Foetal weight measurements were taken, analysis of uric acid and protein levels in placental tissue was conducted, alongside H & E.	Pregnant rats exposed to MSU crystals exhibited decreased foetal weights. The highest dose caused increased placental inflammation, uric acid and IL‐1β protein levels.
Lv et al., 2023 [16]	Primary HUVECs	*n* = 5 for each treatment group: mtDNA, hypo‐mtDNA, hypo‐mtDNA with inhibitor, vehicle.	HUVECs were treated with PBS (vehicle), mtDNA (1 µg/mL), hypo‐mtDNA (1 µg/mL) or hypo‐mtDNA with the NLRP3 inhibitor INF39 (1 µg/mL, 10 µM, respectively). Cells were analysed using flow cytometry.	HUVECs stimulated by trophoblast derived hypo‐mtDNA and mtDNA exhibited significant activation of NLRP3, IL‐1β, caspase‐1, and GSDMD, which was prevented when co‐treated with INF39.
	Murine umbilical cord sera	*n* = 5 for each treatment group of both healthy mice and NLRP3^−/−^ mice: mtDNA, hypo‐mtDNA, hypo‐mtDNA with inhibitor, vehicle.	Both experimental groups of healthy mice and NLRP3^−/−^ mice were treated with PBS (vehicle), mtDNA, (100 µL of 20 µg/µL) or hypo‐mtDNA (100 µL of 20 µg/µL). ELISAs were conducted on the cord serum.	Trophoblast mtDNA increased IL‐1β levels in cord serum, and hypo‐mtDNA increased the pro‐inflammatory response. Cord serum from NLRP3^−/−^ mice had significantly reduced IL‐1β levels compared to WT mice, even when treated with trophoblastic hypo‐mtDNA.
Ozeki et al., 2022 [38]	NLRP3^−/−^ (by CRISPR/Cas9‐mediated genome editing) human first‐trimester Sw.71 trophoblasts	*n* = 4 for both normal trophoblast cell line and NLRP3^−/−^ trophoblast cells.	Trophoblasts were either untreated or stimulated with S100A9 (1 mg/mL for 24 h) and were assessed using an ELISA.	Treatment with S100A9 increased IL‐1β secretion and mRNA expression in the untreated trophoblast cell line and was significantly suppressed in the NLRP3^−/−^ trophoblasts.
	Primary HUVECs	*n* = 3 for normal healthy controls and both treatment groups.	HUVECs were either untreated or stimulated with two different doses of S100A9 (0.5 and 1 mg/mL for 24 h) and analysed using WI.	When stimulated by S100A9, HUVECs demonstrated elevated NLRP3 protein expression.
	Murine placental tissue, plasma and foetuses	*n* = 5 for treatment group, *n* = 4 for healthy control pregnancies.	Treatment groups: S100A9 (20 ng/5 mL) or saline (control) Foetal weights were measured at GD17. An ELISA was conducted on maternal plasma and flow cytometry was conducted on placental tissue.	No changes in foetal weight were found between the groups. IL‐1β concentrations were undetected. The proportion of placental CD45^+^ immune cells and neutrophils was significantly higher in the S100A9 treatment group.
	Human maternal blood plasma	EOPE *n* = 20, LOPE *n* = 30, healthy control pregnancies *n* = 10.	ELISA.	S100A9 levels were increased in both PE groups compared to healthy control pregnancies.
	Human term placental tissue	Samples used for RT‐qPCR and ELISAs: PE *n* = 12, healthy control pregnancies *n* = 6. Samples used for WI: PE *n* = 3, healthy control pregnancies *n* = 3. *n* = 4 for all treatment groups assessed.	RT‐qPCR, ELISA and WI. Treatment groups: S100A9 (0.1, 0.5, 1 mg/mL for 24 h) or control groups with or without NLRP3 inhibitor MCC950 or caspase‐1 inhibitor. Analysed using ELISA and IF.	S100A9 mRNA expression, S100A9 and IL‐1β secretion, and NLRP3 protein levels were all increased in PE placentas compared to healthy control pregnancies. S100A9 stimulation increased IL‐1β secretion in a dose‐dependent manner. When treated with either inhibitor, this secretion was significantly reduced. S100A9 treatment also stimulated caspase‐1 activity and ASC‐speck formation, which was inhibited by MCC950.
Stødle et al., 2018 [69]	Maternal serum	Healthy control pregnancies *n* = 43, PE *n* = 34, non‐pregnant controls *n* = 28.	Measurement of uric acid levels.	Women with PE demonstrated significantly elevated uric acid levels compared to healthy pregnancies and non‐pregnant controls.

Abbreviations: ELISA, enzyme‐linked immunosorbent assay; EOPE, early onset preeclampsia; FGR, foetal growth restriction; H&E, haematoxylin and eosin staining; HUVEC, human umbilical vein endothelial cells; Hypo‐mtDNA, mtDNA obtained from hypoxia‐treated trophoblasts; IF, immunofluorescent staining; LOPE, late onset preeclampsia; mtDNA, mitochondrial DNA; PE, preeclampsia; RT‐qPCR, real‐time quantitative reverse transcription polymerase chain reaction; WI, western immunoblotting; WT, wildtype.

Most investigations rely on LPS‐ and poly I:C‐treated animal models to simulate inflammation during pregnancy [50], exemplified by Alfian et al., who treated trophoblast organoid cultures with LPS and observed increased NLRP3 and caspase‐1 protein expression [14]. LPS treatment of an in vitro trophoblast‐derived cell‐line, BeWo, similarly demonstrated elevated NLRP3, caspase‐1, IL‐1β, and IL‐18 mRNA and protein expression [14]. Nevertheless, this model is not reflective of the sterile inflammation seen in inflammatory placental pathologies. Baker and coworkers have, however, produced a model of sterile inflammation in placental explants [56] and Brien et al. developed a non‐infectious inflammation model using MSU crystals, a form of uric acid (Figure 1), to stimulate cytotrophoblasts and generate responses similar to those observed in FGR [50] (Table 2). These studies demonstrate the link between the NLRP3 inflammasome, sterile inflammation and inflammatory disorders of pregnancy.

Collectively, evidence suggests contributions from NLRP3 inflammasome‐mediated sterile inflammation in the pathogenesis of inflammatory pregnancy disorders. With further investigations, well‐characterised DAMPs could act as endogenous biomarkers for identification of pregnancy complications resulting from placental inflammation [8], and could act as potential therapeutic targets.

## 8 Modulating the NLRP3 Inflammasome Using Drugs and Inhibitors

Inflammatory disorders in early pregnancy are currently treated with non‐steroidal anti‐inflammatory drugs (NSAIDs) [82], including ibuprofen, diclofenac [83], aspirin [84], and fenamic acids [10]. Low dose aspirin (LDA) increases the likelihood of live birth in women presenting with low‐grade inflammation and a previous pregnancy loss [84]. Aspirin's role as an NLRP3 inhibitor has been previously discussed [85], with confirmation that LDA downregulates HMGB1, and subsequently GSDMD, NLRP3, and caspase‐1 [86], suggesting its potential to treat the sterile inflammation in FGR, VUE, and CHI. This is supported by recommendations to take LDA before 16 weeks’ gestation to lower the risk of PE and FGR [84]. However, NSAID use is not advised post 32 weeks’ gestation due to early ductus arteriosus occlusion risk in the foetus [82], prolonged gestation, and antiplatelet activity [87]. The fenamates, mefenamic acid and flufenamic acid, inhibit the NLRP3 inflammasome in rodent models [88]; however, the limited use of NSAIDs throughout pregnancy [82] has restricted their development as treatments for placental inflammation. Opposing this, one study synthesized fenamate analogues which were shown to specifically inhibit the NLRP3 inflammasome, unlike their counterparts, thereby reducing the off‐target effects [89], and with further research could prove as an alternative. Betamethasone therapy postpartum was also trialled in preterm FGR infants, but failed to reduce morbidity and mortality [90], exemplifying the lack of reliable remedial options for placental inflammation.

The NLRP3 inflammasome is a therapeutic target for many diseases outside pregnancy, which has spurred investigations on other small molecule inhibitors. Previous research on direct inhibitors focussed on BAY 11–7082, 3,4‐methylenedioxy‐β‐nitrostyrene, CY‐09, tranilast and Novel Boron Compounds (NBC), which have been studied in context of a variety of diseases [10]. Glibenclamide (previously used to treat GDM) [91] has demonstrated promising results by reducing IL‐1β levels in trophoblast [71] and caspase‐1 protein expression in the supernatants of placental cultures after exposure to oxidative stress [63]; however, its development is limited. In LPS and ATP primed Hofbauer cells, KN‐62 (a P2×7 receptor antagonist), VX765 and WEHD (caspase‐1 inhibitors) all significantly reduced IL‐1β secretion [72], highlighting the range of targets linked to the NLRP3 inflammasome which need to be considered when assessing drug suitability. The NLRP3 inhibitor GDC‐2394 reached human trials for coronary heart disease; however, two participants sustained grade 4 drug‐induced liver injury, thus halting testing [92]. Despite this, Li et al. recently discussed the development of NLRP3 inflammasome inhibitors ZYIL1, OLT‐1177, and RRx‐001 in clinical trials, as well as triazinone and pyridazine compounds preclinically [93], which, if successful, can be explored further.

Research into NLRP3 inflammasome inhibitors using in vitro models may also be advantageous in finding treatments for inflammatory disorders of pregnancy. In a trophoblast model of PE, metformin inhibited pyroptosis through TLR4/NF‐κB signalling via the NLRP3 inflammasome [94]; however, the model relied on LPS‐stimulated inflammation, rather than the sterile inflammation observed in pregnancy. Furthermore, tranilast reduced elevated NLRP3 expression to a greater extent than metformin in placentas from GDM murine models [95]. As a preventative measure, a PE study in humans suggest to monitor and lower MSU levels, thereby targeting the NLRP3 inflammasome [96].

When a PE rat model was treated with the well characterized NLRP3‐inhibitor MCC950 (Table 3), alongside esomeprazole, placental NLRP3 mRNA expression decreased [53], suggesting their roles as potential treatments for placental inflammation. MCC950 also reduced preterm birth and neonatal mortality rates caused by sterile inflammation in murine models [97]. However, MCC950 has also demonstrated adverse effects on the liver, thus limiting its systemic use in pregnancy or any other therapeutic area. Additionally, despite mice sharing haemochorial placentation with humans [98], caution should be heeded in extrapolating results in mice to humans [99].

**TABLE 3:** Potent small molecule and biological inhibitors of the NLRP3 inflammasome.

Name of inhibitor	Structure	Information
**Potent small molecule inhibitors of the NLRP3 inflammasome**		
**MCC950** (cytokine release inhibitory drug 3, CRID3)	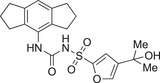	A sulfonylurea compound [10]. Direct potent and selective inhibitor that binds to the NOD domain of NLRP3, targeting its ATPase activity, causing direct inhibition of NLRP3, preventing its activation and IL‐1β production [10, 11, 64, 101, 108]. Efficacy: IC_50_ of 8.1 nM in human monocyte‐derived macrophages [108], IC50 of 7.5 nM in BMDMs [10]. Development is limited due to renal and hepatic toxicity [108].
**NP3‐562**	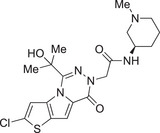	Discovered by Novartis Pharma. Direct inhibition of NLRP3, by binding to the NACHT domain, thus inhibiting NLRP3 activation and IL‐β release [109]. Efficacy: IC_50_ of 214 nM, or 30 mg/kg dose in a mouse model of acute peritonitis [109]. Demonstrated great potency in human whole blood [109].
**NP3‐253**	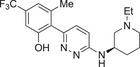	Discovered by Novartis Pharma. Direct inhibition of NLRP3, thus inhibiting NLRP3 activation and IL‐β secretion [110]. Efficacy: IC_50_ of 8 nM, or 2.5 nM in LPS/ATP‐stimulated 50% mouse whole blood, and 0.3 mg/kg in a mouse model of acute peritonitis [110]. Promising potency, physicochemical and pharmacokinetic properties, including brain penetration [110].
**NT‐0796**	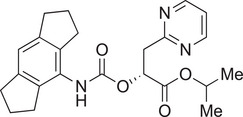	A potent, selective inhibitor that inhibits the release of IL‐1β and IL‐18 [111]. Efficacy: IC_50_ of 0.39 nM, **or** 4.7 µM in human whole blood [111]. Met safety regulations and is currently going through clinical trials to treat neuro‐inflammatory diseases [111].
**Biological inhibitors of the NLRP3 inflammasome**		
**Rilonacept (Arcalyst)**	C_9030_H_13932_N_2400_O_2670_S_74_	An indirect inhibitor dimeric protein that binds to and inhibits IL‐1β [10]. Half‐life: 7 days [10]. Used for treatment of cryopyrin‐associated periodic syndromes and recurrent pericarditis; however, it is administrated via injection [10].
**microRNA as post‐transcriptional inhibitors**	NA	miR‐30a is a direct inhibitor and was shown to inhibit NLRP3 by binding to its 3’UTR [68]. Discovered in model of rheumatoid arthritis [68].

*Note:* Some of these inhibitors are commercially available. Structures were drawn using ChemDraw 23.1.1. software.

Natural products have also been studied as potential NLRP3 inflammasome inhibitors including flavonoids, phenols, and terpenes [10]. Terpenes include parthenolide [10] which targets caspase‐1 and ATPase activity [100], and oridonin [10], which binds to the NOD domain of NLRP3, causing direct inhibition of NLRP3 [101, 102]. Other compounds include brazilin, which directly inhibits priming and activation of the NLRP3 inflammasome and is used in foods; however, further investigations on its toxicity still need to be undertaken [103], and tanshinone I, which has demonstrated favourable effects on septic shock and non‐alcoholic steatohepatitis in mice [104]. Studies on natural products in the context of reproductive health have also been conducted. For example, polyphenol‐rich olive leaf extract (OleaVita) (full composition unknown) reduced IL‐1β secretion, and pro‐IL‐1β and NLRP3 protein expression in human placental tissue [105]. Similarly, the antioxidant L‐ergothioneine reduced IL‐18 and IL‐1β production in placental explants from pregnancies complicated by GDM [106]. In 2024, Jianpi Antai (composition unknown) improved foetal survival rates through inhibition of the NLRP3 inflammasome in spontaneous abortion mouse models [107]. Biological inhibitors provide an alternate method of NLRP3 inflammasome inhibition, with some demonstrating benefits in other disease models. However, research in both natural products and biologics as treatments for placental inflammation is lacking.

Although there are a number of anti‐inflammatory compounds and NLRP3 inhibitors, they lack suitable potency and selectivity (due to infection risk) as well as the necessary pharmacokinetic properties [10], of which none have successfully progressed into clinical trials to treat sterile inflammation‐induced pathologies of the placenta [97]. Highly potent (nM IC_50_ values) small molecule inhibitors of NLRP3 have recently been discovered (Table 3) by NodThera and Novartis pharmaceutical companies. It will be of significant interest to test these compounds in models of placental inflammation.

## 9 Conclusion

FGR and PE are important complications of pregnancy that increase the risk of stillbirth. Placental dysfunction is a major cause of FGR, PE, and stillbirth of which there are likely to be multiple underlying pathologies, including those rooted in sterile inflammation such as VUE and CHI. If unregulated, the NLRP3 inflammasome elicits an inflammatory response instigating various diseases, with recent studies demonstrating its link to the sterile inflammatory pathway. Although limited research has been conducted regarding a potential role of the NLRP3 inflammasome in the pathology of FGR and stillbirth, other placental pathologies with a similar inflammatory profile have suggested a role for the NLRP3 complex. That being said, current literature is inconsistent, and further research is required to fully understand the role of the NLRP3 inflammasome throughout normal pregnancy, and how its expression and regulation change in placental dysfunction. Establishing the link between the NLRP3 inflammasome, sterile inflammation, and inflammatory placental pathologies is crucial, prior to exploration of NLRP3 as a therapeutic target. A lack of effective treatments for FGR and CHI drives the need to develop targeted and efficacious anti‐inflammatory agents, with the ultimate aim of reducing the severity of FGR and the incidence of stillbirths.

## Ethics Statement

The authors confirm that the ethical policies of the journal have been adhered to. No ethical approval was required as this is a review article containing no original research data.

## Conflicts of Interest

The authors declare no conflicts of interest.
